# Where are the hotspots and coldspots of landscape values, visitor use and biodiversity in an urban forest?

**DOI:** 10.1371/journal.pone.0203611

**Published:** 2018-09-26

**Authors:** Silviya Korpilo, Joel Jalkanen, Tarmo Virtanen, Susanna Lehvävirta

**Affiliations:** 1 Ecosystems and Environment Research Programme, Faculty of Biological and Environmental Sciences, University of Helsinki, Helsinki, Finland; 2 Digital Geography Lab, Department of Geosciences and Geography, University of Helsinki, Helsinki, Finland; 3 Department of Landscape Architecture, Planning and Management, Swedish University of Agricultural Sciences, Alnarp, Sweden; Helmholtz-Zentrum fur Umweltforschung UFZ, GERMANY

## Abstract

Cities and urban green areas therein can be considered as complex social-ecological systems that provide various ecosystem services with different synergies and trade-offs among them. In this article, we show that multiple stakeholder perspectives and data sources should be used to capture key values for sustainable planning and management of urban green spaces. Using an urban forest in Helsinki, Finland as a case study, we incorporated data collected using public participation GIS, expert elicitation and forest inventories in order to investigate the guidance that the different types of data, and their integration, can provide for landscape planning. We examined the relationship and spatial concurrence between two social variables i.e. visitors’ perceived landscape values and green space use, and two ecological variables i.e. forest habitat quality and urban biodiversity, using hot/coldspot analysis. We found weak correlations and low mean spatial coincidence between the social and ecological data, indicating great complementary importance to multi-criteria decision-making. In addition, there was a higher level of spatial agreement between the ecological datasets than between the social datasets. Forest habitat quality and urban biodiversity were positively correlated and spatially coincided moderately, while we found a negative correlation and very low overlap between visitor use and landscape values. This highlights the conceptual and spatial distinction between the general preferences and values citizens assign to public green spaces and the realized everyday use of these areas and their services. The resulting maps can inform planners on overall social and environmental quality of the landscape, and point out potential threats to areas of high ecological value due to intensive recreational use, which is crucial information for natural resource management. In the end, we discuss different strategies for managing overlaps and discrepancies between the social and ecological values.

## Introduction

The social-ecological systems (SES) concept views people as an integral part of nature, with social and ecological systems inextricably linked through regular interactions [1–3]. SES are dynamic and complex systems that include various patterns of use, technology, and resource demands [2]. These patterns produce feedbacks that operate within and between multiple levels (subsystems), and at different spatial, temporal and organisational scales [1,2]. The modern city is a key example of a human-dominated SES [4,5]. In this context, an urban forest or a green area can be understood as a social-ecological system or subsystem, where biophysical and social factors regularly interact and produce various ecosystem services (ES) [6,7] shaped by patterns of land use. In order to effectively manage and preserve urban forests and green spaces, while balancing between multiple and possibly conflicting objectives and demands of various stakeholders, planners and managers require good understanding of the social and ecological components of the system, their interactions, and the synergies and trade-offs between the different ES they provide [8–14]. However, the links and spatial dynamics between the social and ecological aspects of urban ES are still insufficiently explored in literature [8]. Particularly, the integration of cultural ES, being generally more intangible and difficult to quantify, has lagged behind in ES mapping and assessments as found in several recent reviews by Crossman et al. [15], Egoh et al. [16] and Haase et al. [8]. In this article, we use Helsinki’s Central Park as a case study to demonstrate how multiple social and ecological data gathered using public participation GIS (PPGIS), expert elicitation and forest inventories can be combined to broaden the perspectives and knowledge base used in integrated assessment approaches. We analyse the supply side of ES through urban biodiversity indicators and the demand for cultural ES in terms of how citizens evaluate (e.g. aesthetic values and opportunities for recreation) and directly use the green space and its services [17]. The overall aim was to investigate the guidance that the different types of data, and their integration, can provide for sustainable spatial planning of multifunctional landscapes.

However, translating heterogeneous and interdisciplinary knowledge about ES into clear and practical recommendations for decision-makers remains challenging, thus, hindering implementation in urban planning and policy [8,18]. For this purpose, we used GIS analysis as an effective way to visualize and integrate the social and ecological variables [3,19] and aid landscape planners by providing understandable spatially-explicit information [20,21]. Hot/coldspot mapping was conducted to analyse the relations between the different data and identify areas with spatial overlaps and discrepancies of high and low social-ecological values [2,10,22]. As compared to previous studies using hotspot analysis in spatial prioritization and conservation planning (e.g. [2,10,12,13,23–25]), our approach adds visitor flows to inform planning and management on potential threats to specific areas with high ecological value due to intensive recreational use [25,26]. More specifically, we investigated the relations and spatial concurrence between landscape values, i.e. multiple perceived values that citizens assign to different areas [2,22,27,28], ecological values (measured by forest habitat quality and urban biodiversity attributes) and visitor use (smartphone GPS-tracked and digitally-drawn visitor routes). Based on the findings, we propose different strategies for managing spatial overlaps and discrepancies between the social and ecological values.

## Materials and methods

### Study area and data inputs

**Fig 1:**
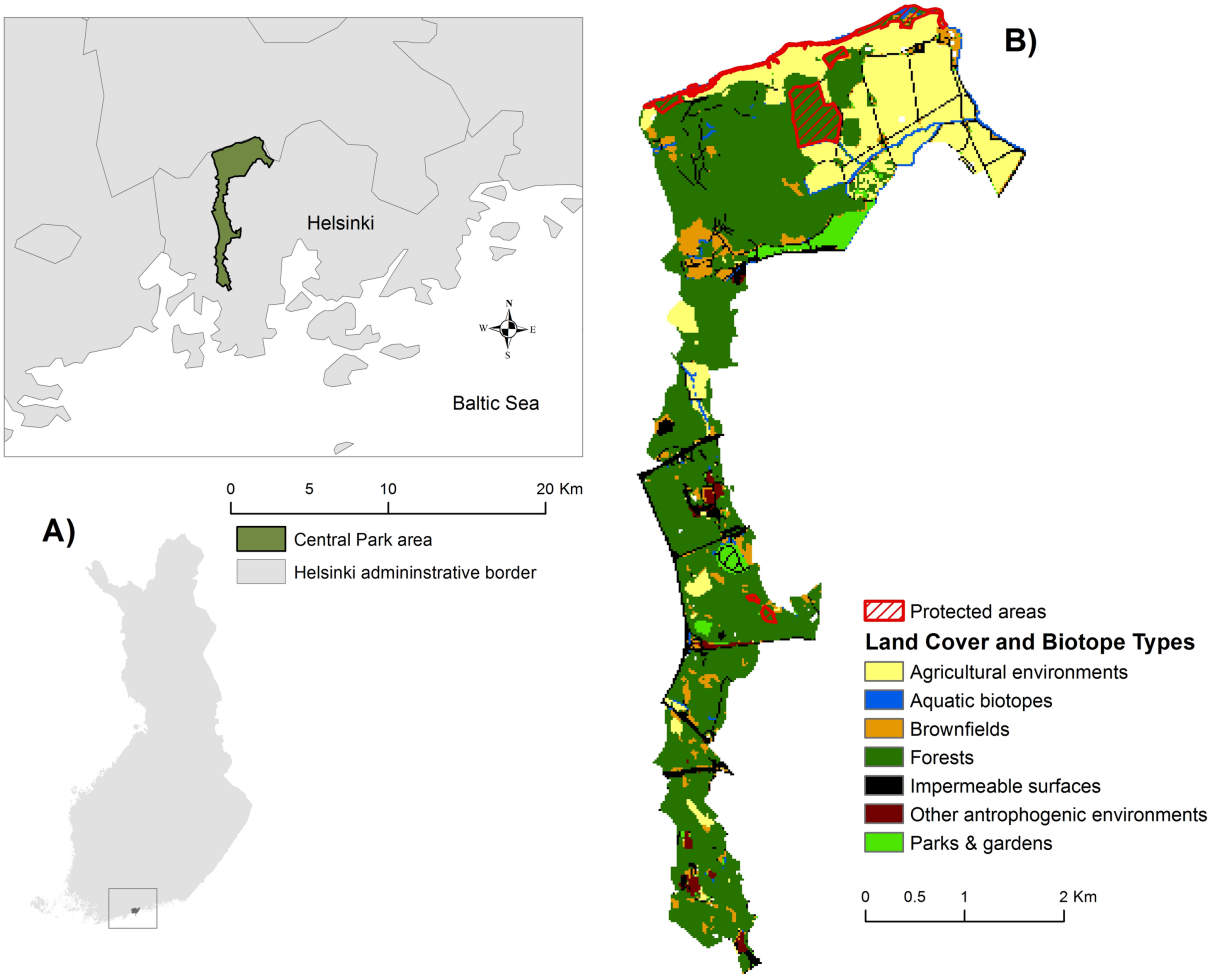
(A) Area and location of Central Park in Helsinki, Finland. (B) Main land cover and biotope types and location of protected areas in Central Park. For detailed list and description of all biotopes, see section Urban biodiversity and S2 Table.

Central Park is the largest single green area and part of the “Green Fingers” network in Helsinki, Finland. It stretches over 10 km all the way to the northern border of the city (Fig 1A). The park covers 1100 ha of mostly forested land, but it also includes other habitats like fields, rocks and rivers, making it an excellent representative of the rich and varied nature and wildlife in the Finnish southern coastal region [29]. There are four nature protection areas in the northern part of the park: two riverside groves, an old-growth forest, and an arboretum (Fig 1B) [29]. Central Park is intensively used for sports and recreation. It includes several sports centres and around 100 km of paths and trails, which are used throughout the year for various activities such as walking, cycling, running, mountain biking, exploring nature, dog walking, commuting, and skiing in the winter [30].

Four types of spatially-explicit social and ecological data were imported in ArcGIS (v.10.2.1) for analysis (Table 1). The social data was collected from two public participation studies, while the ecological data was acquired via expert elicitation and from municipal forest inventory datasets. The datasets were collected for diverse research and planning purposes, using various methods and spatial scales. Data on citizen perceived landscape values were the most spatially coarse including 82 pre-defined areas (hereafter polygons) (see section Social value mapping). To keep the analysis feasible and applicable to planning, we adopted these polygons as a measurement unit for all data analyses since they were defined as useful by the local urban forest planning department.

**Table 1:** Social and ecological data inputs used in the study.

Data inputs	Dataset	N resp.*	Analysis units	Measurement
***Social data***				
Landscape values	On-site and postal visitor survey (2007–2009)	599	Seven positive landscape values, 82 pre-defined polygons	Numeric: count of times a landscape value was assigned per polygon
Visitor use	GPS-tracked and drawn routes collected via web-based PPGIS study (2015)	233	366 tracks (line features)	Numeric: density of tracks (length in m/ha) per polygon
***Ecological data***				
Forest habitat quality	Municipal forest inventory data (2010, 2014)	-	1172 forest management stands	Numeric: sigmoidal function of average tree diameter (m/ha) x wood volume (m^3^/ha) per polygon
Urban biodiversity	Expert elicitation via an online questionnaire (2016)	24	Seven biodiversity attributes, ten taxonomic groups, 37 biotopes	Scale**: mean expert scoring (0–4) weighted by self-rated confidence score (0–8) per polygon

*N resp. = number of respondents

** The scale used by the experts represents scoring each biotope for each of the seven biodiversity attributes of 10 taxonomic groups and level of confidence in their answers (see section Urban biodiversity).

### Social value mapping

#### Landscape values

The landscape value mapping was part of a visitor use survey conducted by City of Helsinki, Public Works Department, in 2007–2009 using on-site and postal methods [31]. The on-site survey was carried out in 2007 and 2008 by students from the University of Helsinki. The fieldwork included 2–3 hours of interviewing each time during October and November. Different areas of the park were covered in the two different years following a purposive maximum variation sampling design: the interviewers were positioned on pre-planned strategic places inside the park, choosing passers-by who appeared to represent different age, gender and activity type. In April 2008 and 2009, the postal survey was sent to local residents in the surrounding areas of Central Park. Both years, the survey was sent to 1000 residents randomly sampled by the Finnish Population Register Center. The survey design was based on previous surveys conducted by Helsinki Planning Department for other areas in Helsinki Metropolitan Region e.g. the center of Espoo and East Helsinki [32]. The questions gathered information on visitor age, gender, use of the park (e.g. activity type, frequency of use) and landscape values.

Respondents were given a paper map of Central Park with pre-defined and numbered polygons with sizes varying between 0.6 ha and 83 ha (66% <10 ha). These areas were identified by city officials primarily based on their local forest management system, however, in some cases, the boundaries were modified so that areas could be better recognised and understood by the general public. A list of landscape values was presented and respondents were asked to write down the number(s) of the polygons to which the value was applicable. The values were briefly described and illustrated with drawings. The list included seven positive values: “scenic view”, “valuable nature site”, “feeling of forest”, “feeling of space and freedom”, “history and culture”, “peace and quiet”, and “opportunity for activities” (for detailed description, see S1 Table). Respondents could assign multiple landscape values to any polygon. Each of the 82 polygons was allocated a count of the times a polygon was identified as containing a specific landscape value. Then, a multiple landscape value was measured per polygon based on the sum of counts for each of the seven landscape values.

Although in general larger polygons are likely to receive a greater number of landscape value scores than smaller ones, in our dataset, each polygon was primarily uniform (i.e. includes a single homogenous landscape type) and distinctive from other polygons in the surrounding areas. For example, a relatively small polygon can consist entirely of an archery range, a dog park or allotment gardens, and it is highly unlikely that increasing the size of such polygons will increase the cumulative multiple landscape value. In addition, our preliminary analysis (using Spearman correlation coefficients) showed that the relation between landscape values and polygon size was mostly statistically insignificant or weak and explaining little of the variance. Therefore, polygon size was not taken into account in the calculations.

#### Visitor use

As part of a collaboration project with City of Helsinki, data on visitor use in Central Park was gathered via a web-based PPGIS study in 2015 (July–December) to inform the new management plan of the area [33]. A web-based tool called “MyDynamicForest” (www.mydynamicforest.fi) was used to collect questionnaire and route data (GPS-tracked and digitally-drawn routes, see [34]). Study participants could submit their route to the MyDynamicForest website in two different formats: 1) by adding a self-tracked GPS route that they had already collected using any sports tracking application (e.g. Sports Tracker, Strava, Endomondo) on their personal smartphones; or 2) by drawing it digitally over an Open Street Map basemap. After submitting a route, participants were asked to answer a short questionnaire that examined their socio-cultural background, activities and route-choice motivations [33,35].

The study was conducted with principles of informed consent and ethical considerations were addressed according to the National Advisory Board on Research Ethics in Finland. Participants were asked to sign a Letter of Consent, which provided clear terms and conditions of voluntary participation. All information was processed as anonymous and aggregated before analysis. Since there may be privacy issues related to using smartphone GPS tracking data [36,37], the routes were cut to fit the borders of the study area and only intra-site tracks were used in the analysis in order to avoid possible tracing of citizens e.g. to their home or work location [33,35].

The collected route data was used in the analysis as representative of actual visitor use. Data cleaning was conducted by removing identical GPS points at the same location fix (e.g. due to participants standing still) and deleting drawn routes that were visually too coarse to be informative [33]. Then, in order to calculate visitor use per polygon, all tracks (as line features) were intersected with the polygons and the total length per polygon was calculated to represent the density of tracks per ha.

#### Respondents’ background

Since the landscape values and visitor use data were collected as part of separate surveys, different categorizations were used to analyse the socio-cultural background of respondents. To allow for comparison, we present the data using as close as possible categories.

A total of 599 respondents took part in the visitor survey conducted by City of Helsinki [31]. The response rate for the postal surveys was 20.8% (208 responses) in 2008 and 21.7% (217 responses) in 2009, while 174 citizens participated in the on-site survey (2007–2008). The gender ratio was relatively balanced (F:M ratio 57.6%:42.4%) and the predominant age group was > 50 years-old (53.4%), while 28.7% were 30–50 years old and 16.8% were < 30 years-old. The respondents were mostly local residents and frequent park users, visiting the park at least once a week (51.4%) or at least once a day (17.6%). Most frequently mentioned activities (including multiple activities per respondent) included walking (75.1% of all responses), cycling (56.3%), running (43.4%), skiing (34.1%) and dog walking (16.7%).

In total 233 respondents took part in the “MyDynamicForest” PPGIS survey during the six-month data collection period, providing 366 tracks (139 GPS-tracked and 277 drawn). Also in this study, the gender ratio was quite even (F:M ratio 45.9%:54.1%), however, the middle age group 35–54 years-old was the largest (50.9%), while 38.1% were < 34 years-old and 11.1% were > 55 years-old [33]. Respondents were again mainly local residents and very frequent park users with 60.1% visiting the park at least once a week and 24.5% visiting the park at least once a day. The route data included running (33.3% of all tracks), cycling (31.4%), walking (13.9%), dog walking (11.5%) and mountain biking (9.8%), representing summer and autumn use of the park.

### Ecological value mapping

We estimated the ecological value of different areas in Central Park using two approaches: 1) measuring forest habitat quality based on forest inventory data [38]; and 2) assessing urban biodiversity value based on expert elicitation. Both methods rely on surrogate data to measure biodiversity i.e. features that act as proxies for species and habitats [38].

#### Forest habitat quality

Forest inventory data can provide detailed information on compositional indicators that directly measure biodiversity (e.g. number of plant species in a specific area) and structural indicators based on key structural features as surrogates for biodiversity [39]. For example, many highly specialized and threatened species in Finnish boreal forests depend on specific resources such as old trees and deadwood [38,40,41]. This study uses structural indicators because such data were available from Helsinki’s regional forest inventories. In addition, structural features might play an important role in planning and decision-making as they are relatively easy to measure [39,42] and could be more robust than e.g. presence of some specialist/indicator species [43]. In addition, they may be easily understood by forest managers who are used to working with forest inventory datasets [38].

Based on expert workshops with the Finnish Forest and Park Service (responsible for the management of the state-owned forest property in Finland) and Finnish Forest Center (advising forest owners across Finland), Lehtomäki et al. [38] developed a *habitat quality index* as a proxy for ecological value. The index uses forest inventory data as an input in the following formula:

$$I_{i}\;=\;f(diameter)\times volume$$

where *I* stands for index value for a cell in location *i*, and *f* is a specific sigmoidal function of the average diameter (an indicator for maturity) multiplied by volume [38]. The forest inventory data for Central Park (2010; 2014), provided by City of Helsinki, was at stand level with sizes varying between 0.1 ha and 11 ha. First, a raster map (20x20 m cell size) was created based on the habitat quality index for each forest stand. Then, Zonal statistics was used to calculate a mean value per polygon representing forest habitat quality value per ha.

An important methodological consideration is that the forest habitat quality index represents a simple function that refers only to forest biotopes and notably gives more value to mature forests with large diameter trees, therefore, other forest types (e.g. spruce mires) or habitats (e.g. agricultural fields) can receive low values [38]. With the urban biodiversity method presented below, we aim to complement this approach by estimating value not only for forested areas but for all biotope types in Central Park, including different kinds of private and public, as well as man-made and natural ecosystems (from e.g. private gardens to brownfield biotopes).

#### Urban biodiversity

As part of a study conducted in the Helsinki Metropolitan Area in 2016 [44], 24 local experts evaluated independently via an online questionnaire how well various urban biotopes support different taxonomic groups: *vascular plants*, *polypores*, *fungi (other than polypores)*, *birds*, *bats*, *mammals (other than bats)*, *herpetofauna*, *butterflies*, *hymenoptera*, and *beetles*. The experts were from the Finnish Museum of Natural History, University of Helsinki, Finnish Environment Institute, and local environmental NGOs, all of which with renowned expertise of these taxonomic groups. The study used written informed consent and all data were aggregated before analysis.

Two to three experts per taxon answered how well each biotope type supports different community attributes of their specific taxon of expertise. They scored from 0 to 4 (from no support to very high support) seven biodiversity attributes per biotope (Table 2; for detailed description of the biodiversity attribute questions, see [44]). For example, if “dry meadow” supports very high species richness of butterflies, a butterfly expert would score that biotope as “4”, while another expert might score dry meadow’s support for polypores as “0”. The compilation of seven attributes was guided by ecosystem functioning and conservation principles (e.g. [45–48]). To facilitate respondents, the questionnaire included a description of all biodiversity attributes and biotope classes used in the study (see [44]).

**Table 2:** Biodiversity attributes used in the expert questionnaire.

Biodiversity attribute	Description
Species richness*	How greatly does the biotope support species richness of the focal taxon?
*Specialist species*	To what extent does the biotope support specialist species of the focal taxon?
*Biomass*	How large is the combined biomass of all individuals of the focal taxon in the biotope?
*Abundance*	How great are the numbers of individuals of the focal taxon in the biotope?
*Evenness*	How evenly are the numbers of individuals of the focal taxon distributed across different species in the biotope?
*Uniqueness*	Are there such species assemblages in the biotope that are not found in other biotopes?
*Representativeness*	How representative or “high-quality” (high species richness or diversity, rare species, etc.) are species assemblages found in the biotope compared to other similar or identical biotopes in Southern Finland?

*Terms such as “species richness” were not specified but used as open, general concepts.

In addition to the scoring, experts gave confidence ratings to their answers corresponding to “very unconfident”, “unconfident”, “somewhat unconfident”, “somewhat confident’” and “very confident”, which were given a value of 0, 1, 2, 4 and 8 in the analysis. Therefore, the scores could theoretically produce values from 0 (a biotope is scored among all experts as having no support for the different taxa, and/or responses were given with very low confidence) to 32 (very high support of the biotope and very high confidence in the responses).

We created a raster biotope map of Central Park consisting of 38 different biotopes that followed the biotope classification used in the questionnaire (a simplified map with major biotope types is shown in Fig 1B; for detailed list of biotopes and their description, see S2 Table). The map (20x20 m cell size) was based on the forest inventory data and an urban biotope map for the whole Helsinki Metropolitan Area [49]. We then assigned the mean confidence-weighted scores to each biotope class. Raster values for biotope classes were determined as the average of the scores given by experts for each taxonomic group for the focal biotope, weighted by the self-rated confidence. Then, Zonal Statistics were used to calculate value per polygon, representing an overall mean expert score as an average across all biotopes and taxa within that polygon.

### Comparing and integrating the social and ecological data

**Fig 2:**
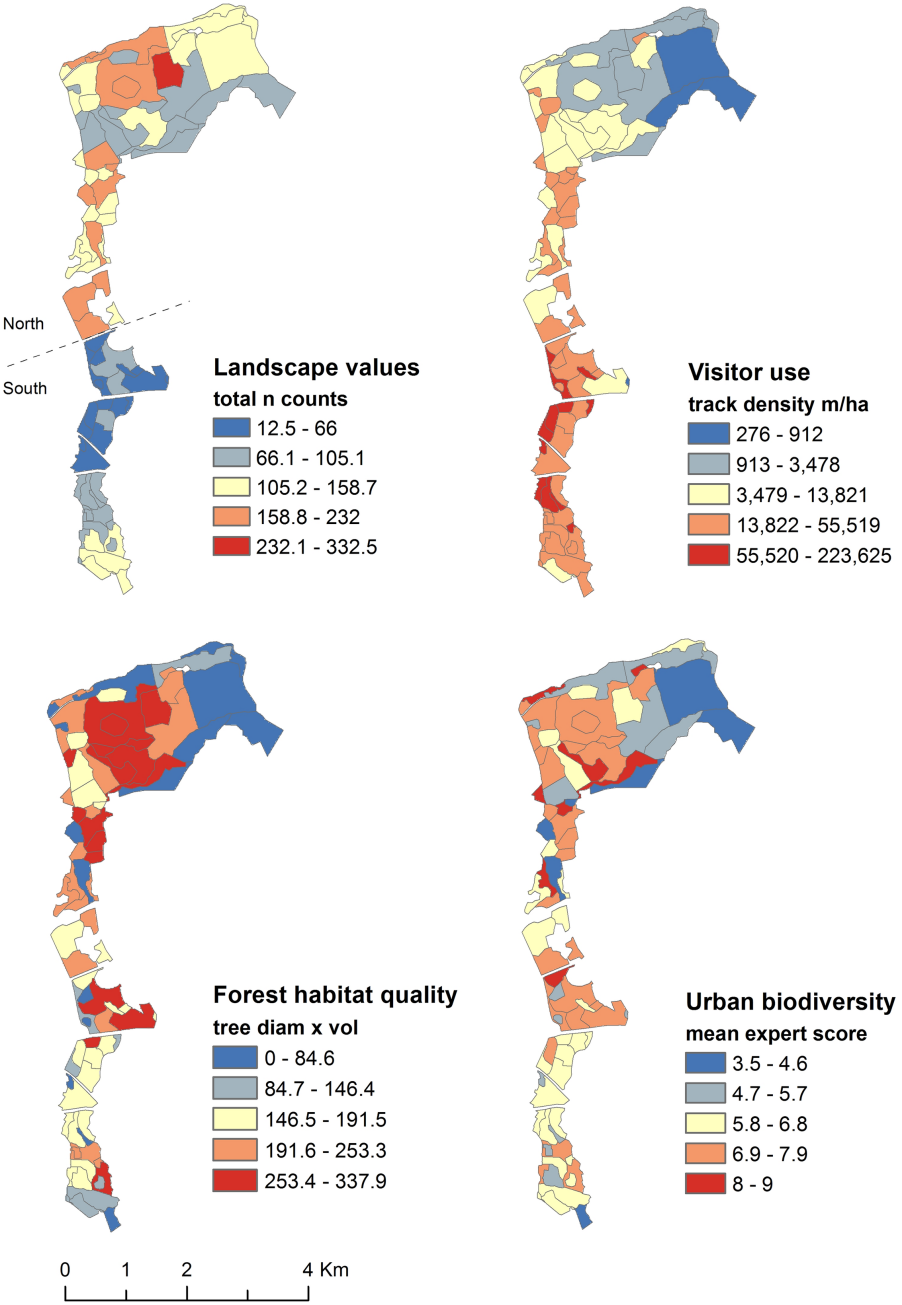
Distribution of social (landscape values, visitor use) and ecological (forest habitat quality, urban biodiversity) values in Central Park.

All data were imported in SPSS (v 21.0) and normalized by converting each value range from 0 to 1 so that the individual datasets were given equal weight in the analysis [10]. Pairwise correlations between the datasets were analysed using Spearman correlation coefficients. In order to determine whether there were underlying factors affecting the spatial distribution of landscape values and visitor use, we conducted additional analysis with population density in the surrounding areas and density of formal (managed) trails within the park. Proximity to green space is often associated with increased use with a common threshold of 300-1000m (15 min walk) of distance decay [50–52]. Therefore, to investigate potential links between high visitor use and population density, we calculated density of inhabitants per km^2^ (population data from [53] within 300 m and 1000 m from the border of the study area. The estimations were done separately for the southern and the northern part of the park, following Pirkkolantie road as a division point (see Fig 2). In addition, following the same approach as for visitor use density, we calculated trail density (trail network from [54]) per ha for each of the 82 polygons. The resulting values were used to test the correlations (using Spearman correlation coefficient) between landscape values, visitor use density and trail density.

Then, hot/coldspot mapping was conducted for each dataset, as well as for all datasets combined, in order to locate areas of spatial overlap between high and/or low ecological and social values. To define the hot/coldspots in this study, we used a common quantile delineation method [2,12,24,55–57], which divides the data into classes with an equal number of cells. Quantile sizes often vary between 5 and 30% [13] and here, we used the upper and lower third of the value range (top 33% defined as hotspots and bottom 33% as coldspots). This generous cut-off was considered most appropriate for the analysis because our data were based on relatively large pre-defined polygons and smaller delineation percentages resulted in insufficient outputs e.g. areas too small to be informative for landscape decision-making.

The spatial concurrence between hot/coldspot areas was estimated using the Jaccard coefficient [24,58,59]. The Jaccard coefficient directly measures spatial overlap between each pair of social and ecological hot/coldspots, producing values from 0 to 100, where a larger Jaccard coefficient indicates greater spatial overlap [24]. The Jaccard coefficient (J) was calculated using the following formula:

$$J\;=\;\frac{Number\;ofhot\;or\;coldspot\;grid\;cells\;shared\;by\;the\;two\;layers}{Number\;of\;additional\;grid\;cells\;belonging\;to\;the\;two\;layers}\times \;100$$

Here, we define Jaccard coefficient values as follows: 0–20% = very low overlap, 20–40% = low, 40–60% = moderate, 60–80% = high, and 80–100% = very high overlap. Then, social-ecological hot/coldspot mapping was performed to identify areas of spatial synergies and discrepancies between high and low values in Central Park [56,58]. We overlaid the combined social with the combined ecological value i.e. the summed and equally weighted multiple landscape values and visitor use with the summed and equally weighted forest habitat quality and urban biodiversity value, respectively. In addition, we compared hot/coldspots of visitor use with landscape values, and visitor use with the combined ecological value. Then, following a similar approach as in Whitehead et al. [12] and Schröter et al. [26], we outlined practical recommendations for managing areas with overlaps and discrepancies between the different values.

## Results

### Spatial distribution of values and their correlations

Among all respondents to landscape value survey, the most frequently mapped values included “feeling of forest” (69.4% of respondents), followed by “valuable nature site” (62.8%), “scenic view” (61.1%), “space and freedom” (57.6%), “peace and quiet” (54.3%), “opportunity for activities” (47.6%) and “history and culture” (37.4%). The summed multiple landscape value per polygon ranged from 12.5 to 332.5 and highest values were assigned to areas in the northern part of Central Park (Fig 2). At the same time, the density map of visitor use (n = 366 route tracks) portrayed an opposite spatial distribution to the landscape values, with the highest density of use located in the southern part of the park (Fig 2). In line with the visitor use spatial patterns, population density was higher around the southern part of Central Park both within a 300/1000 m distance from the park border (4185/4300 inhabitants per km^2^ in the south, 1287/1861 in the north).

The forest habitat quality index produced values ranging from 0.0 to 337.9 (Fig 2). Highest values were derived for older forest habitats including mature (30–100 yrs) and old-growth (> 100 yrs) heathland forests, spruce mires, and herb-rich forests, while non-forested habitats like agricultural fields, golf courses and shrubby wastelands produced the lowest values. The urban biodiversity expert scores ranged from 3.5 to 9.0 (Fig 2) and similarly to the forest habitat quality results, highest scores were assigned to mature and old-growth heathland forests and herb-rich forests, while lowest values were located in e.g. agricultural fields, golf courses and heavily managed built parks. However, differences in the value range could be observed when comparing the maps in detail. For example, Haltiala nature reserve, which comprises of old-growth heathland forests and spruce mires, received the highest score for forest habitat quality (normalized value = 1.0), whereas its mean expert score for urban biodiversity was in the top mid-range (0.76). Conversely, a fresh meadow area received a high value for urban biodiversity (0.81) while scoring very low for forest habitat quality (0.07).

The inverse relationship between landscape values and visitor use was further highlighted by the statistically significant negative correlation between these datasets (Table 3). When analysed individually, all landscape values were negatively and significantly correlated with visitor use, except “feeling of forest” (-0.131, *p* = 0.240) and “valuable nature site” (-0.105, *p* = 0.357), which showed no significant relationship. Perhaps not surprisingly, trail density and visitor use density indicated a significant positive correlation (0.526, *p =* <0.001). At the same time, no significant correlation was found between landscape values and trail density (0.101, *p* = 0.365). Forest habitat quality and urban biodiversity were positively and most strongly correlated among all data (Table 3). For all other pairwise comparisons, we found no significant correlations.

**Table 3:** Spearman correlation coefficients between the social and ecological values.

Pairwise correlations	*Correlation coefficient*	*p*
Landscape values and visitor use	-0.421	<0.001
Landscape values and forest habitat quality	0.195	0.080
Landscape values and urban biodiversity	0.064	0.567
Visitor use and forest habitat quality	-0.035	0.758
Visitor use and urban biodiversity	0.075	0.502
Forest habitat quality and urban biodiversity	0.626	<0.001

### Socio-ecological hot/coldspot mapping

The multiple comparisons of hot/coldspot overlap using the Jaccard coefficient indicated a low mean coincidence between the social and ecological data of 23.9% for the hotspots and 30.0% for the coldspots. The highest overlap was observed between the two ecological variables i.e. forest habitat quality and urban biodiversity (50.4% in the hotspots and 61.5% in the coldspots). The overlap between landscape values and visitor use hotspots and coldspots was very low (Table 4). There was also very little overlap between the citizen-mapped landscape values and expert-driven urban biodiversity value, while visitor use and the ecological variables showed moderate similarity between the coldspots and very low to low coincidence between the hotspots (Table 4).

**Table 4:** Degree of spatial overlap for hot/coldspots based on Jaccard coefficient (%) between the social and ecological data.

	Visitor use	Forest habitat quality	Urban biodiversity
Landscape values	8.6/5.7 (hot/coldspot)	26.3/14.9	20.4/12.2
Visitor use		12.7/39.6	25.3/46.2
Forest habitat quality			50.4/61.5

**Fig 3:**
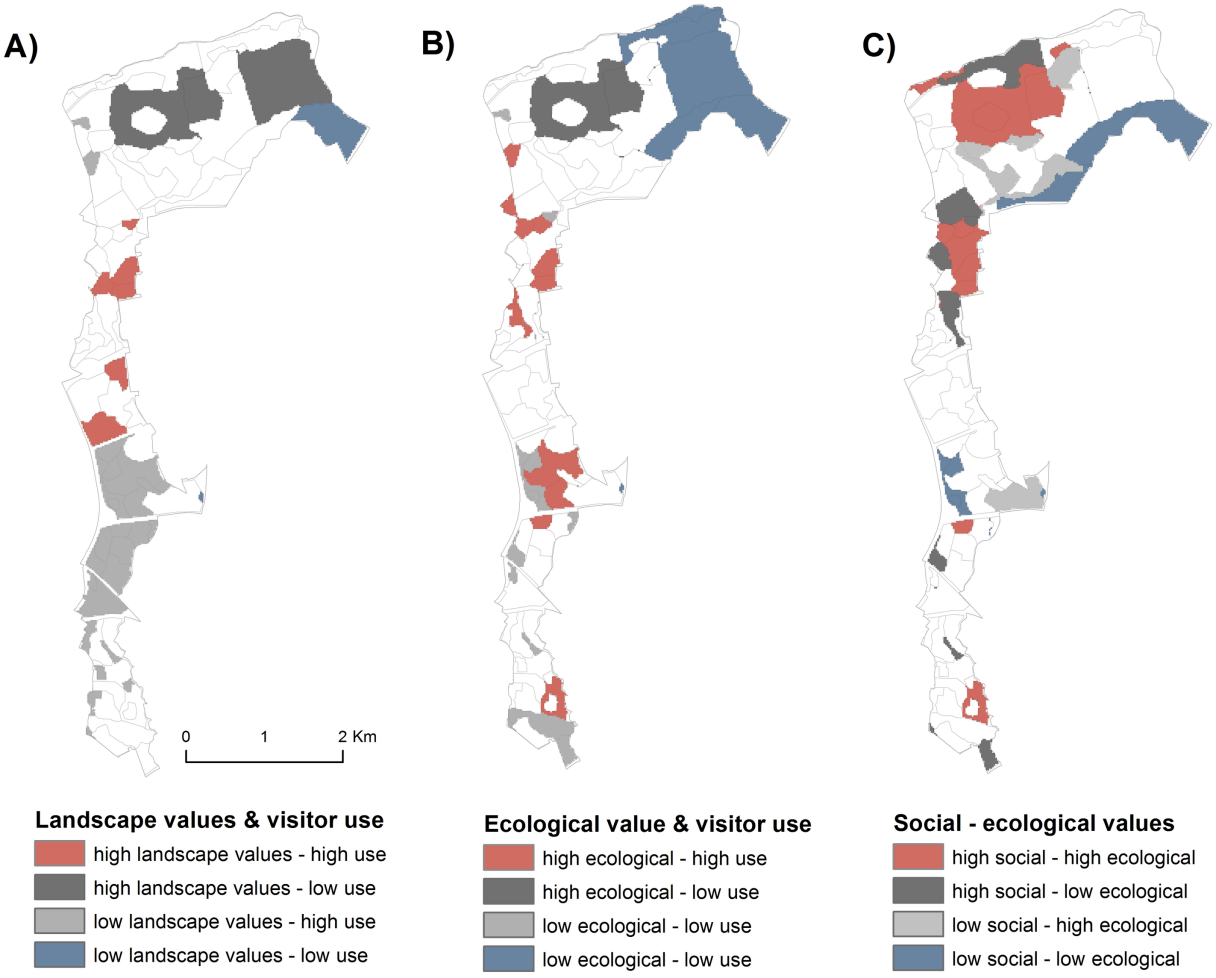
Hot/Coldspot pairwise overlays. (A) Multiple landscape values and visitor use, (B) Combined ecological value and visitor use and (C) Combined social value and combined ecological value. High and low areas represent top and bottom 33% of values for each metric respectively.

The overlay of visitor use and landscape values generated relatively small total area of hotspots (43.6 ha) and coldspots (33.3 ha) (Fig 3A). It also showed notable discrepancies with a large total area of low landscape values and high visitor use (167.24 ha), and vice versa (142.0 ha). The visitor use and ecological value coldspots covered an area of 177.8 ha, mostly in the agricultural fields in the northeast part of the park (Fig 3B), while the hotspots were observed in more localities, but covered a smaller total area of 81.2 ha. Yet, a different spatial distribution of high and low values resulted from the integration of the combined social and combined ecological values (Fig 3C). The total hotspot area, representing both high ecological and high social importance, increased to 153.6 ha, which was much larger in size than coldspot areas with overlapping low values (93.8 ha).

## Discussion

Our examination of the spatial relations between the different social and ecological data suggested two important findings. First, we found only low mean spatial concurrence between the social and ecological values (23.9% for the hotspots and 30.0% for the coldspots) in Helsinki’s Central Park, which builds upon the results of others reporting up to 30% spatial overlap in ES hotspot mapping (e.g. [2,12,24,60]). The low spatial overlap together with lack of significant correlations and high variation between the social and ecological data suggests that these datasets are of high complementary importance to multi-criteria decision-making [24]. In addition, tapping into multiple sources of knowledge can make land use planning generally more inclusive and socially-acceptable [2,12,19,58].

Second, there was a higher level of spatial agreement between the ecological datasets than between the social datasets. Forest habitat quality and urban biodiversity were significantly and positively correlated and indicated an overall moderate degree of overlap. This implies that these two approaches identify different areas as valuable and their integration can provide a more comprehensive and robust assessment of biodiversity in diverse landscapes. Further, planners and managers can take advantage of the detailed spatial information contained within each dataset as guidance for more targeted decision-making. For example, the raster maps may offer valuable information about the ecological quality of particular stands or biotopes within a hotspot area. An important consideration is that we calculated forest habitat quality based on a generic index across all tree species, although values may vary according to species (e.g. pine, birch, spruce, other deciduous trees) [38]. An advancement of this methodology would also include the amount and diversity of large size deadwood [61,62].

Another important result is that the two social datasets (landscape values and visitor use) showed visually opposite distribution patterns, a negative correlation, and very low overlap between the hot/coldspots (<9%). Such low level of agreement indicates that these variables hold distinctive value and each one can largely influence the mapping, consequently leading to different guidance for decision-making.

The high visitor use density in the southern part of Central Park could be explained by the higher population density in the surroundings, generally narrower shape of the park and higher density of trails, while much of the land in the northern part is covered by agricultural fields, where the public has spatially and temporally limited access. We also expected that high density of trails might negatively affect aesthetic experiences and thus be associated with decreased landscape values. Yet, we found no negative or statistically significant correlation between trail density and landscape values, which suggests that other explanatory factors should be analysed in future research such as users’ perceptions on crowding [63,64]. In addition, an important limitation to this study is the relatively small sample size of the visitor use dataset and the lack of seasonal representativeness. The PPGIS route data was collected only during summer and autumn, while the north part of Central Park is heavily used for skiing in the winter [31]. It is imperative to broaden the sample and collect further data on winter use in order to investigate the potential effect of such data on the density mapping and the degree of overlap with landscape values. It is also worthwhile to further examine the effect of age i.e. whether it makes a difference that the dataset portraying values is based on a sample where more than half of the respondents were >50 years old, while the dataset portraying use was sampled from younger generations. In addition, there was a time difference of six to eight years between the two surveys and more up-to-date data could be gathered to identify possible changes in citizens’ preferences and values. Although, as in this case, we acknowledge that limited resources may not allow for frequent data collection, we recommend that user surveys are updated in line with adapting local management plans (e.g. every ten years).

A key explanation to the low similarity between the social datasets could be that the landscape values did not successfully capture everyday use which was represented by the route data. The list of values given to respondents included mostly aesthetic, cultural and therapeutic values (e.g. scenic view, valuable nature site, feeling of forest, history and culture, peace and quiet) [22,27,65] that represent the general desire or preferences for cultural ES services, while visitor use revealed the realized everyday use of these services [17]. Even recreational value (i.e. opportunities for activities) was negatively correlated with recreational use. This leads us to conclude that although both values and use could be predictors of social importance, they carry different conceptual meaning and represent different aspects of human relationships with the environment. These results highlight the necessity of including both visitor flows and perceived values into hot/coldspot analysis for natural resource planning and management.

Although we found no statistically significant relationship between visitor use and the ecological values, visually inspecting the location of overlaps and discrepancies between these datasets could be well informative for planning at the landscape scale (Fig 3B). The maps can help guide spatial decision-making by locating areas of high biodiversity value and high level of threat from intensive recreational use. Figs 2 and 3B point out that the central part of Central Park is of high concern as intensive use in this area might jeopardize both the ecological and social quality of the forest. Assessing in-situ the degree of wear and ecological impacts in these hotspot areas (e.g. see [66]) can facilitate designing appropriate reactive management strategies [26].

Based on our results and informed by the recommendations of other socio-ecological hotspot mapping studies [12,26], Table 5 below is our effort to make the synergies and trade-offs between different ES explicit and provide examples of science-based guidelines for managing multiple and conflicting elements. While Table 5 is not intended to be an exhaustive list, the aim is to show that strategies could emphasize different issues and be planned and implemented with different levels of citizen engagement depending on the specific context. For effective interpretation of the guidelines, the hot/coldspots should be understood as a continuity rather than a dichotomy [10], and so should be the management implications. The different cells should not be seen as restrictive but rather as interlinked and adaptive processes. Urban SES are dynamic and strategies should consider the changing environment. For example, due to the rapidly changing climate, urban forests in Helsinki more frequently lack snow cover during winter [67]. Access ways like duckboards could freeze and become dangerous and unfit for recreational use, leading to widening of paths on often very sensitive forest floor vegetation [68]. Therefore, the location and e.g. choice of materials requires careful planning and co-design with users as part of adaptive forest management.

**Table 5:** Examples of strategies for managing synergies and discrepancies between landscape values, visitor use and the combined ecological value based on the hot/coldspot mapping.

		High ecological value	Low ecological value
**High landscape values**	**High visitor use**	Recognise and maintain key ecological and landscape elements, and monitor visitor use; consider reactive conservation in most ecologically degraded sites; high potential for citizen engagement in monitoring and maintenance	Recognise and maintain key valuable landscape elements; study potential areas of conflicts (e.g. crowding, conflicts between user groups) and ways to mitigate them; potential for engaging the public in ecological restoration
	**Low visitor use**	Study reasons for low use; aim for spatially limited use e.g. improve access to selected areas with subtle, spatially-concentrated access ways (e.g. duckboards, high walkways); enhance public awareness of the ecological value	Study the reasons for low use; use can be encouraged and guided here from other close-by areas to alleviate pressure (potential conflict resolution areas), while maintaining valuable landscape elements; consider regeneration/restoration of ecological value
**Low landscape values**	**High visitor use**	Potential areas of concern; monitor and manage effects of recreational use; high potential for recruitment of users for planning; co-design attractive landscape elements along main routes to encourage visitors to stay on formal paths	Study the reasons for low landscape values; high potential for user conflict and conflict resolution; high potential for recruitment of users for co-design
	**Low visitor use**	Sites with high potential for proactive conservation approaches; natural dynamics and conservation targets could be the main focus of planning and maintenance	Potential sites for reconciliation ecology and experimental society; use co-planning and experiments to improve quality

### Methodological implications

The choice of social and ecological variables included in the analysis, as well as the integration methods [56], has important implications for the ES hot/coldspot mapping results. First, in survey-based research, the wording and presentation of questions can easily affect responses [69]. Here, the drawings used in the landscape values survey (see S1 Table) may have been misleading as also suggested by Tyrväinen et al. [32], who studied social values of urban forests in East Helsinki using a similar questionnaire. For example, “feeling of space and freedom” was portrayed as a field (see S1 Table), which could result in a confirmation bias. Feeling of space and freedom does not necessarily relate to openness, instead, it could be understood as perception of moving freely and experiencing no boundaries [52]. Despite these limitations, high diversity of citizen values assigned to a specific area is very likely to reflect a high social value of that area [57].

Similar considerations are relevant to the expert questionnaire used to measure urban biodiversity as the questions were presented on a very general level. For example, “uniqueness” may be very difficult to measure objectively or to incorporate into a question that every individual will interpret in the same way: some species may be associated with urban regions, however, it remains challenging to determine when a species assemblage becomes unique.

Second, data in this study were collected as part of different research projects, and at different time and spatial scales. Generally, our study shows that using pre-defined polygons can offer a viable and systematic way for integrating heterogeneous social and ecological datasets. However, using relatively large polygons provides less detailed spatial information compared to point-based mapping, and low resolution may increase the probability of spatial errors, e.g. missing high values of small areas [70,71]. PPGIS mapping can be also biased towards familiar places, potentially discarding value of important but less familiar locations [71]. In addition, including negative experiences and values (e.g. fear and unpleasantness), which are beyond the scope of this study, could help better identify areas of low value (coldspots) (see e.g. [32]).

Further, we used a generous quantile method (top/bottom third) for defining the hot/coldspots, which also involves some trade-offs. A large quantile cut-off may compile very high or low value areas with moderate high or low value areas within a single hot or coldspot [56]. Therefore, it is recommended to always investigate how the chosen scale and delineation method affect actual values in a dataset, and understand the consequent implications. Here, the resulting maps were presented to forest planners from City of Helsinki and the level of detail was found sufficient and consistent with the scale used in the new nature management plan for the area.

## Conclusions

Urban planning and management needs to balance between high pressure on urban green spaces caused by multiple and often competing land use demands, densification policies, fragmentation and citizens’ need for attractive and accessible green spaces as an integral part of urban liveability. Although the socio-ecological context may differ considerably from one setting to another, the values citizens assign to different landscapes, visitor use, and biodiversity indicators are likely to remain relevant to governance of all public natural resources. In many parts of the world, there are significant political and economic barriers to acquire such data, however, advancements in internet and mobile participatory data collection tools indicate promising directions for future research. We also emphasize that while avoiding deterioration of areas with high ecological and high social quality (i.e. hotspots) is crucial, also the coldspots, i.e. areas of lower quality, should receive further attention in literature and planning processes. This would involve re-defining such areas by minimizing negative social values e.g. fear or use conflicts, or turning heavily-worn and ecologically degraded areas into sites for experimental design [72]. Co-production of knowledge and co-design, and the active role of citizens and researchers from multiple disciplines in improving the quality of such areas should become a norm for planning the landscapes of tomorrow [9,23,72]. It is also crucial to reveal which parts of the target population have been reached, allowing to specifically address the ones who were underrepresented.

## Supporting information

S1 Table Description of landscape values used in Helsinki’s Central Park visitor survey (2007–2009). (PDF)

S2 Table A list of urban biotopes in Helsinki’s Central Park included in the expert questionnaire. (XLSX)
