# Supplementary material for: Where are the hotspots and coldspots of landscape values, visitor use and biodiversity in an urban forest?
Source: PLoS One. 2018 Sep 26;13(9):e0203611. doi: 10.1371/journal.pone.0203611 (PMC6157851; doi:10.1371/journal.pone.0203611)
Supplement: S1 Table — (PDF) [file pone.0203611.s001.pdf]

S1 Table. Description of landscape values used in Helsinki's Central Park visitor survey (2007-2009)

Landscape value	Description
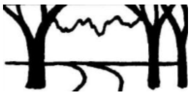 Scenic view	Places or areas that you find beautiful and attractive
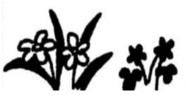 Valuable nature site	Valuable nature area or place with a special feeling of nature (e.g. natural vegetation, animals)
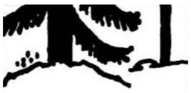 Feeling of forest	Area or place that feels like 'real' forest
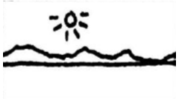 Feeling of space and freedom	Area or place where you can enjoy space and freedom
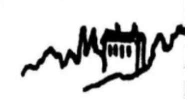 History and culture	Area with interesting local history
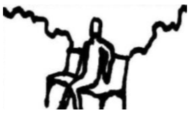 Peace and quiet	Area or place that is peaceful and quiet
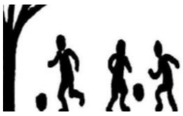 Opportunity for activities	Area with good opportunities for play and recreation (e.g. playfields, outdoor areas, facilities)

Source: Ilvesniemi, S., Saukkonen, T. Keskuspuiston käyttäjätutkimus 2007–2009 (CentralPark Visitor Survey 2007-2009). Public Works Department, City of Helsinki. 2015. Available from: http://www.hel.fi/www/hkr/fi/palvelut/suunnitelmat/keskuspuisto.
